# Integrated Assessment of Physiological, Molecular and Ultrastructural Responses to Heat Stress in Wheat

**DOI:** 10.3390/plants15121896

**Published:** 2026-06-18

**Authors:** Saida T. Zulfugarova, Samira M. Rustamova, Aynura N. Pashayeva, Fuad H. Rzayev, Eldar K. Gasimov, Irada M. Huseynova

**Affiliations:** 1 Institute of Molecular Biology, Ministry of Science and Education of the Republic of Azerbaijan, Baku AZ1073, Azerbaijan; szulfuqarova@icloud.com (S.T.Z.); s.rustamova@imbb.science.az (S.M.R.); aynurapashayeva@gmail.com (A.N.P.); 2 Department of Electron Microscopy, The Scientific Research Center, Azerbaijan Medical University, Baku AZ1078, Azerbaijan; fuad.rzayev@amu.edu.az; 3 Department of Histology, Embryology and Cytology, Azerbaijan Medical University, Baku AZ1078, Azerbaijan; eldar.gasimov@amu.edu.az; 4 Department of Molecular Biology and Biotechnologies, Baku State University, Baku AZ1148, Azerbaijan

**Keywords:** *Triticum aestivum* L., heat stress, membrane stability, antioxidant enzymes, gene expression analysis, Western blot, *HSP16.9*, *DREB*, SOD isoforms, ultrastructural alterations

## Abstract

Heat stress severely constrains wheat productivity, yet the mechanisms underlying thermotolerance remain incompletely understood. This study integrated physiological, biochemical, molecular, and ultrastructural analyses to characterize heat-stress responses in four bread wheat (*Triticum aestivum* L.) genotypes contrasting in heat tolerance. Membrane injury was assessed by membrane damage rate, lipid peroxidation by malondialdehyde accumulation, antioxidant defense by SOD, CAT, GPX, and BPX activities, and stress-responsive regulation by qRT-PCR analysis of *DREB*, *HSP16.9*, and SOD isoforms. HSP16.9 protein accumulation was further evaluated by Western blotting. Heat stress increased membrane damage and MDA accumulation in all genotypes; however, tolerant Murov 2 and Zirva 85 showed lower oxidative membrane injury than sensitive Aran and Gyzyl bugda. Thermotolerance was associated with stronger antioxidant activation, enhanced *DREB* and *HSP16.9* induction, and more coordinated *FeSOD* and *MnSOD* expression. The HSP16.9 protein accumulated after heat treatment, supporting its role as a stress-responsive molecular chaperone. Separate correlation analyses of tolerant and sensitive genotypes revealed stronger coordination among transcriptional, chaperone-related, and antioxidant markers in tolerant genotypes, whereas sensitive genotypes showed a more fragmented response. Microscopy further showed better preservation of chloroplast, mitochondrial, and mesophyll organization in the tolerant genotype relative to the sensitive counterpart, indicating integrated cellular protection. Together, these responses define a coordinated tolerance strategy that may guide the selection of heat-resilient wheat genotypes.

## 1. Introduction

Wheat (*Triticum aestivum* L.) is one of the most important staple crops worldwide, supplying nearly 20% of the global human caloric and protein intake and playing a crucial role in food security [1]. It is cultivated on more than 200 million hectares, with an annual production exceeding 800 million tons [2]. However, wheat productivity is increasingly threatened by climate change, particularly by rising temperatures. The global mean temperature has increased by approximately 0.2 °C per decade and is projected to rise by 2.5–4.8 °C by the end of this century under high-emission scenarios [3], resulting in more frequent and intense heat waves that destabilize crop yields [4]. Each 1 °C increase in temperature can reduce wheat yield by 6–10% [5]. Although the optimal temperature for wheat development ranges between 20 and 23 °C, exposure to high temperatures (>30 °C), particularly during critical reproductive stages including anthesis and grain filling, leads to severe physiological damage and accelerated senescence [6]. To mitigate heat-induced damage, wheat plants activate complex defense systems involving coordinated physiological, biochemical, and molecular responses, including adjustments in photosynthesis, antioxidant activation, and the induction of heat shock proteins (HSPs), which stabilize cellular proteins and prevent aggregation [7]. Under heat stress, a rapid accumulation of stress-responsive proteins, particularly HSPs, occurs, as these proteins act as molecular chaperones that stabilize denatured proteins and protect cellular structures under elevated temperatures [8]. HSPs contribute to proteostasis by preventing irreversible protein misfolding and aggregation and by facilitating downstream protein quality-control processes. The expression of *HSPs* is regulated by heat shock transcription factors (*HSFs*), which serve as central regulators of the heat stress response and activate transcription of heat-responsive genes [9]. Based on molecular weight and conserved domains, plant HSPs are classified into major families—including HSP100, HSP90, HSP70, HSP60, HSP40 (J-domain proteins), and small HSPs (sHSPs)—each with distinct roles in thermotolerance. sHSPs are ubiquitous and highly conserved, binding a broad range of partially unfolded proteins and maintaining them in a foldable state for subsequent rescue by ATP-dependent chaperone systems [10]. In wheat, one of the best-characterized members of this family is TaHSP16.9, a cytosolic class I small heat shock protein with demonstrated chaperone activity that effectively protects thermolabile proteins from heat-induced aggregation [11]. The oligomeric assembly and structural organization of sHSPs have been resolved at high resolution, providing critical insights into their functional architecture and substrate-binding mechanisms [12]. Transcriptomic and proteomic analyses in bread wheat and related grasses indicate that α-crystallin domain-containing sHSPs are strongly induced by elevated temperatures and play a crucial role in maintaining proteostasis and stress adaptation [13]. Moreover, members of the sHSP family in cereals participate in complex chaperone networks that enhance heat resilience under stress conditions [14]. Although much of the available research has focused on the structural organization of TaHSP16.9, its heat-inducible expression further supports its functional role in maintaining cellular proteostasis under thermal stress [15]. Heat stress also disrupts cellular redox homeostasis through the excessive accumulation of reactive oxygen species (ROSs) in multiple subcellular compartments, leading to impaired photosynthetic efficiency, loss of membrane integrity, oxidative injury to plant tissues, and a significant reduction in grain number and weight under prolonged thermal conditions. These effects are associated with the generation of diverse ROSs, including singlet oxygen, superoxide radicals, and hydroxyl radicals, thereby disturbing the delicate balance between ROS production and scavenging [16]. To counteract oxidative damage, plants activate a sophisticated antioxidative defense system composed of both enzymatic and non-enzymatic components that collectively maintain cellular redox homeostasis. This system includes key antioxidant enzymes, such as superoxide dismutase (SOD), catalase (CAT), and ascorbate peroxidase (APX), together with major non-enzymatic antioxidants, including ascorbate, glutathione, carotenoids, and α-tocopherol, which are rapidly activated under heat-induced oxidative stress and play a central role in ROS detoxification and the preservation of cellular integrity [17]. In wheat, multiple SOD isoforms, including *CuZnSOD*, *FeSOD*, and *MnSOD*, constitute key elements of the enzymatic antioxidant defense system that regulate intracellular ROS levels under heat stress, thereby mitigating oxidative damage and maintaining redox balance [18]. Among these isoforms, *MnSOD* has been proposed as a potential biochemical marker for heat tolerance, as its enhanced expression and activity under elevated temperatures correlate with improved protection against ROS accumulation [19]. The coordinated upregulation of these isoforms contributes to enhanced antioxidant capacity, membrane stability, and overall stress resilience [20]. Importantly, the antioxidant network functions synergistically to scavenge excess ROSs and prevent oxidative damage, while ROSs themselves also act as signaling molecules that regulate stress-responsive pathways and enhance plant adaptability under adverse environmental conditions [21]. At the cellular level, biomembranes represent highly sensitive systems and primary targets of thermal injury [22]. Environmental stress factors can induce both specific and nonspecific responses in plants, with nonspecific reactions reflecting a general cellular response to diverse adverse conditions [23]. One of the earliest nonspecific effects of heat stress is the destabilization of cytoplasmic membranes, manifested by increased permeability, protein denaturation, and, in severe cases, cell death [24]. Because membranes function as natural barriers and regulatory interfaces, they are exposed to heat stress earlier than other cellular components, and their destabilization often triggers cascading metabolic disturbances [25]. Membrane thermostability, defined as the ability of membranes to preserve their structural integrity and functional selectivity under stress conditions, is therefore considered a key physiological determinant of heat tolerance in wheat (*Triticum aestivum* L.) [26]. Stress-induced changes in membrane permeability, particularly to electrolytes, are widely used as quantitative indicators of thermal injury and thermotolerance [27]. At the ultrastructural level, membrane destabilization manifests as pronounced disorganization of membrane-rich organelles, particularly chloroplasts and mitochondria. These changes include thylakoid disorganization, swelling of grana, disruption of envelope membranes, and accumulation of plastoglobules, all of which impair photosynthetic efficiency and energy metabolism under high temperatures [28]. The present study aimed to clarify the cellular basis of heat stress tolerance in bread wheat (*Triticum aestivum* L.) by evaluating contrasting genotypic responses to elevated temperature. Rather than focusing on a single protective mechanism, the study considered thermotolerance as an integrated trait shaped by coordinated physiological, biochemical, molecular, and structural adjustments. This integrative perspective was used to identify the main features associated with improved heat resilience in wheat.

## 2. Results

### 2.1. Heat-Induced Membrane Damage and Lipid Peroxidation

**Figure 1:**
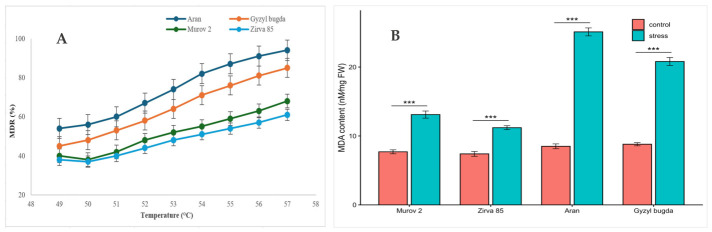
Heat-induced membrane damage and lipid peroxidation in wheat genotypes. (**A**) Membrane damage rate (MDR) in leaf samples exposed to increasing temperatures from 49 °C to 57 °C; (**B**) malondialdehyde (MDA) content in control and heat-stressed leaf samples. Data are presented as mean ± SE (*n* = 3). Asterisks indicate significant differences between control and heat-stress treatment within each genotype: *** *p* < 0.001.

Increasing temperature induced a progressive increase in membrane damage rate (MDR) in all studied wheat genotypes, indicating a gradual loss of membrane integrity under heat stress (Figure 1A). The strongest heat-induced membrane injury was observed in Aran, where MDR increased from approximately 54% at 49 °C to about 94% at 57 °C. Gyzyl bugda also showed pronounced membrane destabilization, with MDR increasing from about 45% to 85% over the same temperature range. In contrast, the tolerant genotypes Murov 2 and Zirva 85 maintained comparatively lower levels of membrane damage. MDR increased from approximately 38% to 68% in Murov 2 and from about 38% to 61% in Zirva 85. A similar temperature-dependent pattern was observed for electrolyte leakage, which is presented in Supplementary Table S1.

Consistent with the changes in membrane stability, heat stress significantly increased malondialdehyde (MDA) accumulation in all investigated genotypes (Figure 1B). Two-way ANOVA confirmed significant effects of genotype, heat treatment, and genotype × treatment interaction on MDA content (all *p* < 0.001; Supplementary Table S2), indicating that lipid peroxidation was strongly dependent on both genotype and heat exposure. Under control conditions, MDA content remained relatively low and varied only slightly among genotypes, ranging from 7.4 to 8.8 nM mg^−1^ FW (fresh weight). Heat exposure led to a marked increase in MDA content, with the highest values observed in the sensitive genotypes Aran and Gyzyl bugda, reaching 25.1 and 22.8 nM mg^−1^ FW, respectively. In contrast, the tolerant genotypes Murov 2 and Zirva 85 accumulated considerably lower amounts of MDA under heat stress, with values of 13.1 and 11.2 nM mg^−1^ FW, respectively. Thus, lower MDA accumulation in Murov 2 and Zirva 85 was consistent with their reduced membrane damage, suggesting more effective protection against heat-induced lipid peroxidation.

### 2.2. Heat-Stress-Induced Expression of HSP16.9 and DREB

**Figure 2:**
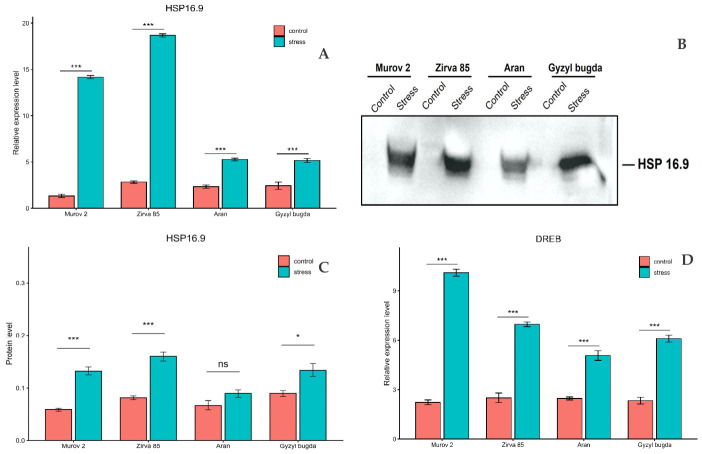
Heat-stress-induced expression of *DREB* and *HSP16.9* in wheat leaves. (**A**) Relative *HSP16.9* transcript levels; (**B**) Western blot of HSP16.9 using an anti-HSP16.9 antibody; (**C**) densitometric quantification of HSP16.9 protein accumulation; (**D**) relative DREB transcript levels. Values are presented as mean ± SE. Asterisks indicate significant differences between control and heat-stress treatment within each genotype: * *p* < 0.05; *** *p* < 0.001; ns, not significant.

Heat stress markedly induced *HSP16.9* transcript accumulation in all wheat genotypes, although the magnitude of induction differed among them (Figure 2A). Two-way ANOVA showed significant effects of genotype, treatment, and genotype × treatment interaction on *HSP16.9* expression (all *p* < 0.001; Supplementary Table S2). The highest fold induction was detected in Murov 2 (10.62-fold), followed by Zirva 85 (6.59-fold), whereas Aran and Gyzyl bugda showed much lower increases, reaching 2.26- and 2.12-fold, respectively. To determine whether these transcript-level differences were also reflected at the protein level, total soluble proteins were separated by SDS–PAGE and transferred to a polyvinylidene difluoride (PVDF) membrane for immunoblot analysis using a polyclonal anti-HSP16.9 antibody. Under control conditions, HSP16.9 protein levels were low and, in some genotypes, produced faint bands below the visual detection threshold; however, basal expression was confirmed by densitometric quantification using ImageJ (version 1.53k; National Institutes of Health, Bethesda, MD, USA). Following heat treatment, HSP16.9 protein accumulated markedly in all genotypes (Figure 2B). Densitometric analysis of band intensities, normalized to total protein per lane, revealed significant stress-induced accumulation in Murov 2, Zirva 85, and Gyzyl bugda, while the increase in Aran did not reach statistical significance (Figure 2C). The strongest protein accumulation was observed in Zirva 85, followed by Murov 2 and Gyzyl bugda, with the weakest response in Aran. Overall, the protein accumulation pattern was broadly consistent with the transcript data and further supported genotype-dependent differences in HSP16.9 induction. In addition, in silico analysis of the *HSP16.9* promoter identified a *DRE*-related cis-element in the upstream regulatory region, providing preliminary support for possible *DREB*-associated transcriptional responsiveness (Supplementary Figure S1). Heat stress also significantly induced *DREB* expression in all four genotypes, although the magnitude of induction differed among them (Figure 2D). Two-way ANOVA confirmed significant effects of genotype, treatment, and genotype × treatment interaction on *DREB* expression (all *p* < 0.001). The strongest increase was observed in Murov 2 (4.52-fold), followed by Zirva 85 (2.79-fold), Gyzyl bugda (2.61-fold), and Aran (2.05-fold).

To provide additional independent support for the heat-responsive nature of the analyzed genes, publicly available wheat transcriptomic data were examined in silico using the Expression Atlas database (EMBL-EBI; dataset E-MTAB-8520 [29]). In this dataset, the *HSP16.9*-related gene TraesCS3D02G045600, annotated in Ensembl Plants/WikiGene as encoding a 16.9 kDa class I heat shock protein 2-like protein, was strongly upregulated under a warm/hot temperature regimen compared with control conditions at both 10 and 14 days post anthesis, with log_2_ fold changes of 5.8 and 4.4, respectively. In the same dataset, the A-genome *DREB*-related gene TraesCS3A02G099200 was also significantly induced under warm/hot temperature conditions at 14 days post anthesis, with a log_2_ fold change of 1.7 (Supplementary Table S3).

### 2.3. Response of Antioxidant Enzymes to Heat Stress

**Figure 3:**
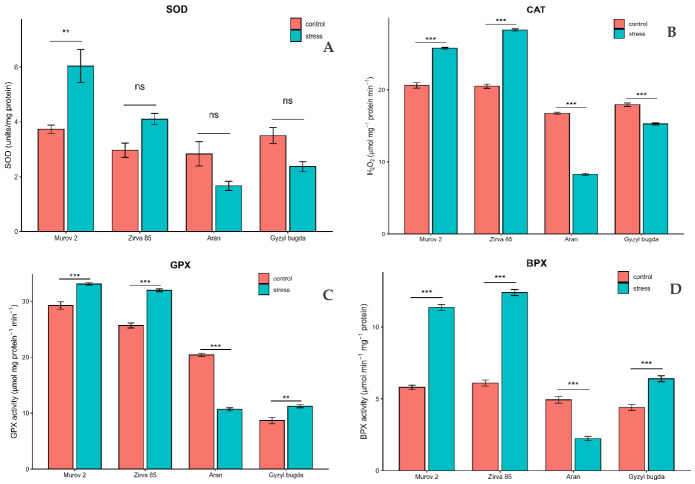
Heat-stress-induced modulation of antioxidant enzyme activities in wheat genotypes. Activities were measured in wheat leaf samples under control and heat stress conditions: (**A**) total superoxide dismutase (SOD); (**B**) catalase (CAT); (**C**) guaiacol peroxidase (GPX); (**D**) benzidine peroxidase (BPX). Values are presented as mean ± SE. Asterisks indicate significant differences between control and heat-stress treatment within each genotype: ** *p* < 0.01; *** *p* < 0.001; ns, not significant.

A clear genotype-dependent pattern was observed in the antioxidant enzyme system under heat stress. Total SOD activity responded differently across genotypes (Figure 3A). Two-way ANOVA showed significant effects of genotype and genotype × treatment interaction on total SOD activity, whereas the overall treatment effect was not significant (*p* < 0.001, *p* < 0.001, and *p* = 0.234, respectively). Under heat stress, SOD activity increased significantly only in Murov 2, reaching 1.62-fold of the control level. In Zirva 85, activity increased to 1.38-fold of the control but without statistical significance, whereas Aran and Gyzyl bugda declined to 0.59-fold and 0.68-fold of their respective control levels and likewise showed no significant changes. A more consistent pattern was observed for CAT (Figure 3B). Two-way ANOVA confirmed significant effects of genotype, treatment, and genotype × treatment interaction on CAT activity (*p* < 0.001, *p* < 0.01, and *p* < 0.001, respectively). Heat stress increased CAT activity to 1.25-fold of the control in Murov 2 and to 1.38-fold in Zirva 85, but reduced it to 0.49-fold in Aran and 0.85-fold in Gyzyl bugda. GPX showed a partly similar but less polarized response (Figure 3C). Two-way ANOVA again revealed significant effects of genotype, treatment, and genotype × treatment interaction (*p* < 0.001, *p* < 0.05, and *p* < 0.001, respectively). Heat stress increased GPX activity in Murov 2, Zirva 85, and Gyzyl bugda to 1.13-, 1.25-, and 1.30-fold of the control levels, respectively, whereas in Aran it declined sharply to 0.52-fold of the control. The largest relative changes were recorded for BPX (Figure 3D). Two-way ANOVA showed significant effects of genotype, treatment, and genotype × treatment interaction on BPX activity (all *p* < 0.001). Heat stress increased BPX activity to 1.45-fold of the control in Gyzyl bugda, 1.96-fold in Murov 2, and 2.03-fold in Zirva 85, whereas in Aran it fell to 0.45-fold of the control level.

### 2.4. Isoform-Specific Expression Patterns of SOD

**Figure 4:**
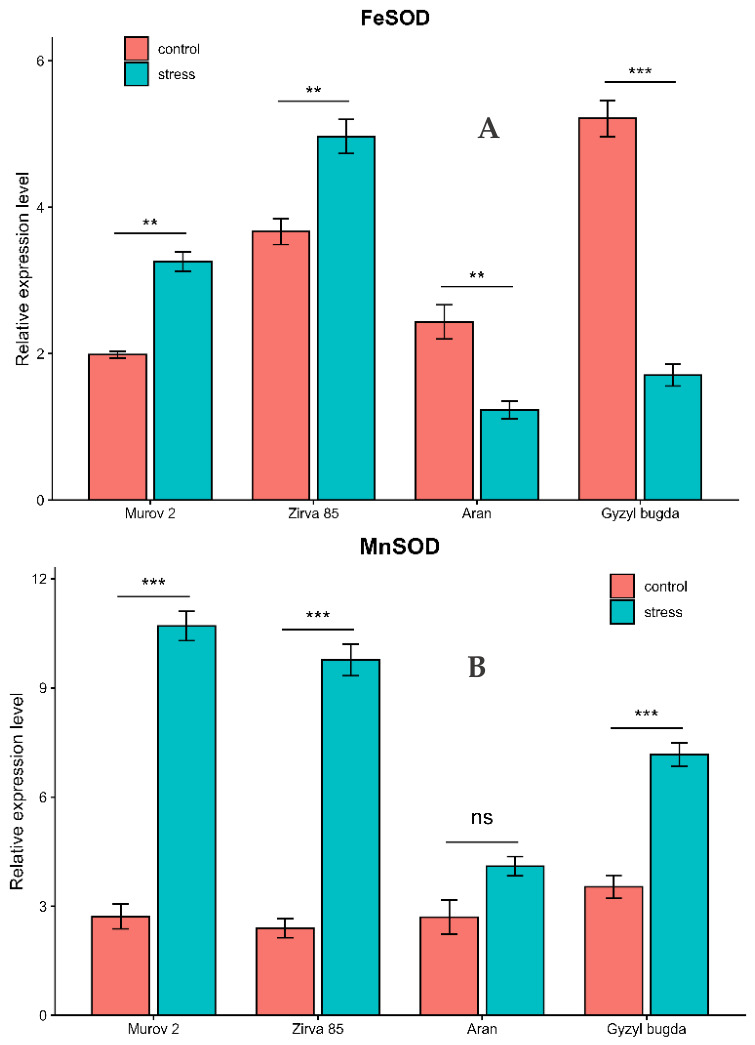

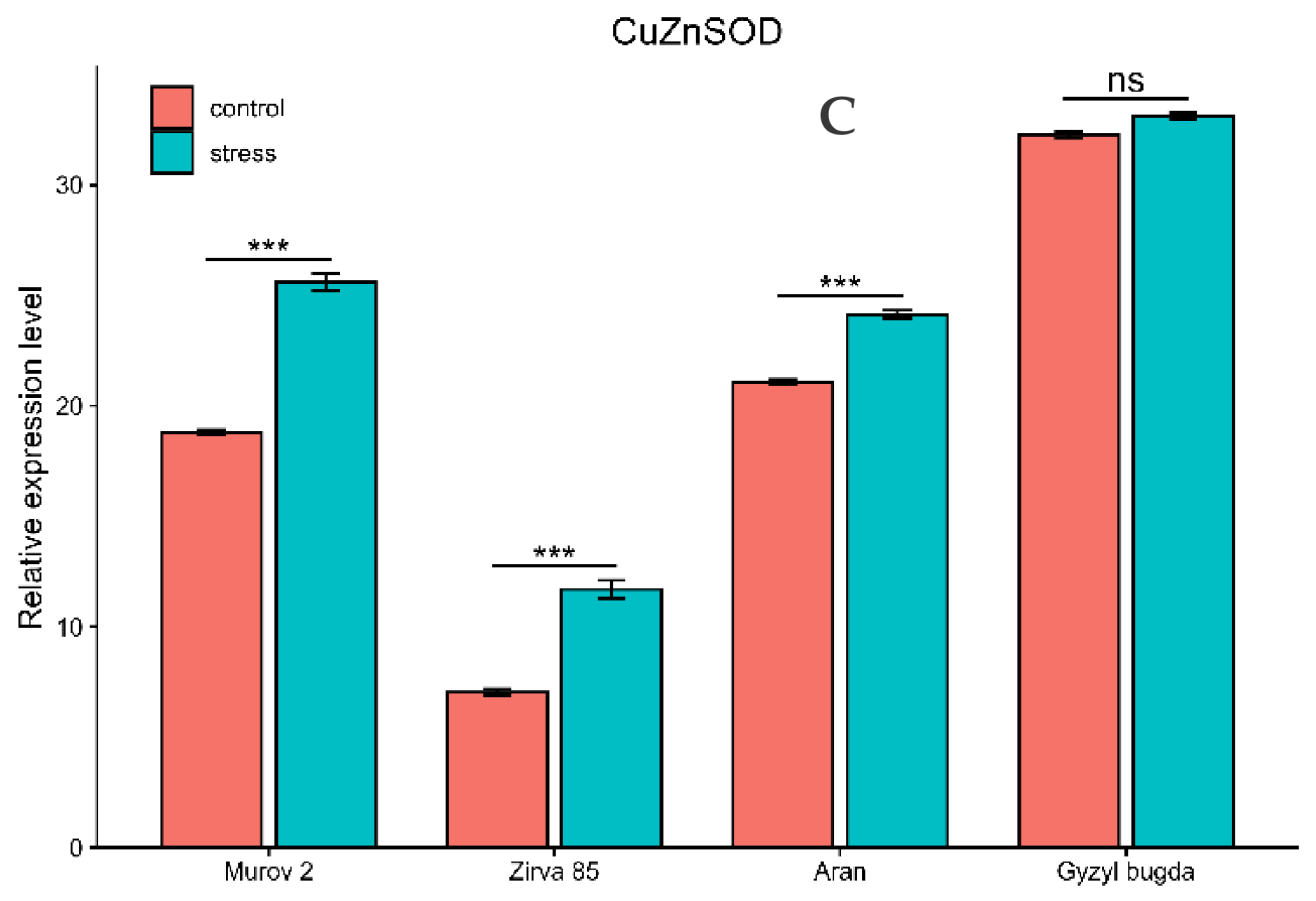
Isoform-specific expression patterns of superoxide dismutase (SOD) genes under heat stress in wheat. Transcript analysis was carried out in control and heat-treated wheat leaf samples. (**A**) iron superoxide dismutase (*FeSOD*); (**B**) manganese superoxide dismutase (*MnSOD*); (**C**) copper/zinc superoxide dismutase (*CuZnSOD*). Values are presented as mean ± SE. Asterisks indicate significant differences between control and heat-stress treatment within each genotype: ** *p* < 0.01; *** *p* < 0.001; ns, not significant.

To assess whether the heat-stress response differed among SOD isoforms, the expression of *FeSOD, MnSOD*, and *CuZnSOD* was analyzed. For *FeSOD*, genotype, treatment, and genotype × treatment interaction all had significant effects on expression (two-way ANOVA, all *p* < 0.001). Heat stress increased *FeSOD* expression in Murov 2 and Zirva 85 by 1.64-fold and 1.35-fold, respectively, but reduced it in Aran and Gyzyl bugda to 0.51-fold and 0.33-fold of the corresponding control levels (Figure 4A). For *MnSOD*, genotype, treatment, and genotype × treatment interaction also had significant effects on expression (two-way ANOVA, all *p* < 0.001). Heat stress increased *MnSOD* expression in Murov 2, Zirva 85, and Gyzyl bugda by 3.93-fold, 4.07-fold, and 2.03-fold, respectively (Figure 4B). In Aran, *MnSOD* expression increased to 1.52-fold of the control level, but this change was not statistically significant. The expression profile of *CuZnSOD* differed from those of *FeSOD* and *MnSOD* (Figure 4C). Genotype, treatment, and genotype × treatment interaction all significantly affected *CuZnSOD* expression (two-way ANOVA, all *p* < 0.001). Heat stress increased *CuZnSOD* expression in Murov 2, Zirva 85, and Aran to 1.36-fold, 1.66-fold, and 1.15-fold of the corresponding control levels, whereas Gyzyl bugda showed no significant change and remained at 1.03-fold of the control level.

### 2.5. Genotype-Specific Correlation Analysis of Integrated Heat-Stress Markers

**Figure 5:**
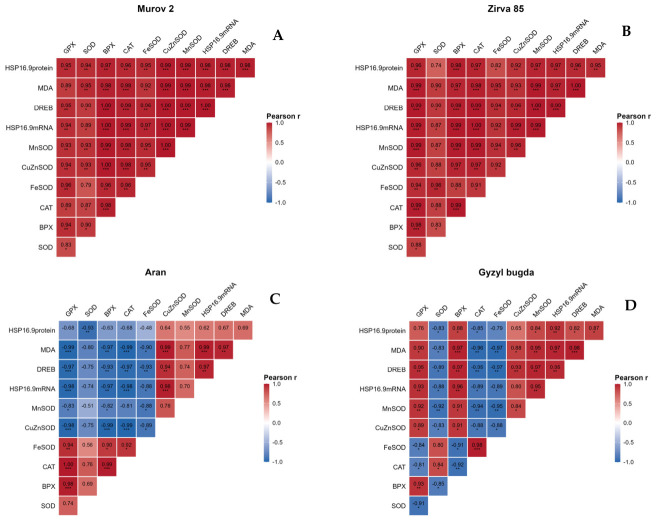
Genotype-specific Pearson correlation matrices of physiological, antioxidant, and heat stress-responsive molecular markers in wheat under heat stress. (**A**) Murov 2, (**B**) Zirva 85, (**C**) Aran and (**D**) Gyzyl bugda. Correlation coefficients (r) between biochemical, antioxidant, and gene expression parameters are represented by color-coded cells. Red indicates positive correlations, whereas blue indicates negative correlations. Statistical significance is denoted as * *p* < 0.05, ** *p* < 0.01, and *** *p* < 0.001. Only the lower triangular portion of the matrices is displayed.

To further clarify genotype-dependent relationships among heat-stress markers, Pearson correlation analysis was performed separately for each wheat genotype: Murov 2, Zirva 85, Aran, and Gyzyl bugda (Figure 5). In Murov 2 and Zirva 85, most analyzed parameters showed positive correlations. In Murov 2, *DREB* expression, *HSP16.9* transcript accumulation, HSP16.9 protein accumulation, *MnSOD, CuZnSOD, FeSOD*, total SOD, BPX, CAT, GPX, and MDA were generally positively correlated with one another (Figure 5A). A similar pattern was observed in Zirva 85 (Figure 5B). In both tolerant genotypes, *FeSOD* and *CuZnSOD* showed strong positive correlations. In Aran, the correlation matrix showed a mixed pattern (Figure 5C). *DREB* expression, *HSP16.9* transcript accumulation, HSP16.9 protein accumulation, *MnSOD, CuZnSOD*, and MDA were positively associated with one another. However, *DREB-* and *HSP16.9*-related parameters showed negative correlations with several antioxidant parameters, including total SOD, BPX, CAT, GPX, and *FeSOD*. In Gyzyl bugda, positive correlations were observed among *DREB* expression, *HSP16.9* transcript and protein accumulation, *MnSOD, CuZnSOD*, BPX, GPX, and MDA (Figure 5D). At the same time, total SOD, CAT, and *FeSOD* showed negative correlations with several *HSP16.9-* and *DREB*-related parameters. Negative associations were also observed among some antioxidant enzyme parameters.

Overall, the genotype-specific correlation matrices showed predominantly positive relationships among physiological, antioxidant, and molecular heat-stress markers in Murov 2 and Zirva 85, whereas Aran and Gyzyl bugda displayed more heterogeneous correlation patterns with both positive and negative associations.

### 2.6. Ultrastructural Changes Under Heat Stress

**Figure 6:**
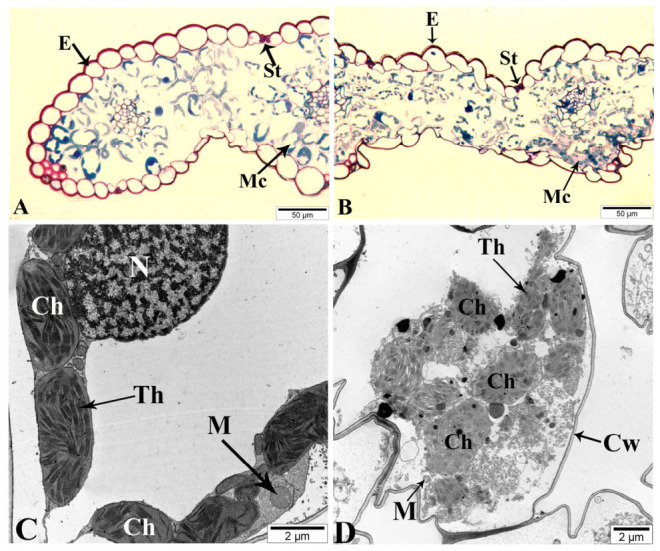
Ultrastructural organization of mesophyll cells in the tolerant wheat genotype Murov 2 under control and heat stress conditions. (**A**,**B**) Light microscopy images; (**C**,**D**) transmission electron microscopy images. (**A**,**C**) Control; (**B**,**D**) heat-treated samples. Ch, chloroplast; M, mitochondrion; Cw, cell wall; E, epidermis; St, stomata; Mc, mesophyll cell; N, nucleus; Th, thylakoid.

**Figure 7:**
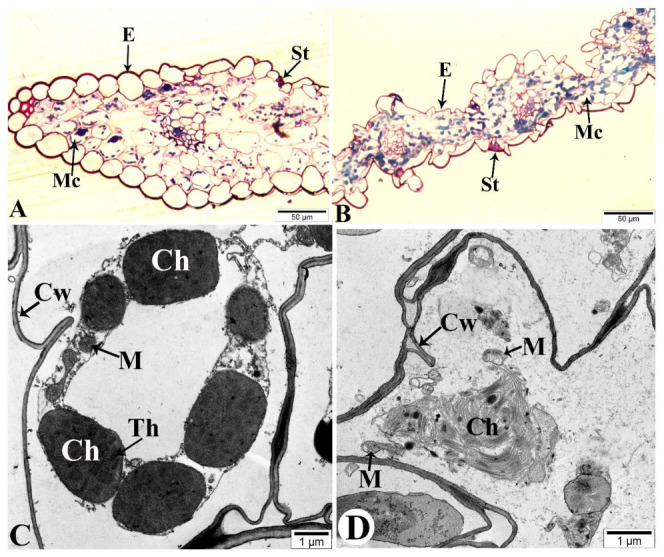
Ultrastructural organization of mesophyll cells in the sensitive wheat genotype Aran under control and heat stress conditions. (**A**,**B**) Light microscopy images; (**C**,**D**) transmission electron microscopy images. (**A**,**C**) Control; (**B**,**D**) heat-treated samples. Ch, chloroplast; M, mitochondrion; Cw, cell wall; E, epidermis; St, stomata; Mc, mesophyll cell; Th, thylakoid.

To examine heat-induced ultrastructural changes in leaf mesophyll cells, two contrasting wheat genotypes, Murov 2 and Aran, were analyzed by light microscopy and transmission electron microscopy. Under control conditions, mesophyll cells of the Murov 2 genotype exhibited a well-preserved cellular organization (Figure 6A,C). A light microscopy image (Figure 6A) confirmed the normal anatomical structure of the plant leaf. The epidermal layer (E) was continuous and compact, while the mesophyll cells (Mc) were well arranged, with clearly distinguishable intercellular spaces. The stomatal region (St) also appeared structurally intact, indicating proper gas exchange capacity. In TEM images, chloroplasts (Ch) were elongated and regularly shaped, with clearly visible and well-organized thylakoid membranes (Th). The chloroplast envelope remained intact, and the stroma appeared homogeneous, indicating a normal functional state of the photosynthetic apparatus. Mitochondria (M) showed a typical oval shape with clearly defined inner membranes and well-developed cristae, suggesting active respiratory metabolism. The nucleus (N) and the elements within it (euchromatin and heterochromatin) are in normal structure. The cell wall (Cw) was intact and uniform, reflecting good mechanical stability of the cells (Figure 6C). After exposure to heat stress, noticeable ultra-structural modifications were observed in the leaf cells of the Murov2 genotype (Figure 6B,D). Light microscopy showed moderate changes in the mesophyll (Mc) structure. Some deformation of mesophyll cells and partial enlargement of intercellular spaces were visible, but the epidermal layer (E) remained largely intact. No structural changes were detected in the stomatal (St) region (Figure 6B). TEM images revealed partial alterations in chloroplast (Ch) ultrastructure, including slight swelling, changes in the organization of thylakoid membranes (Th), and a less compact grana arrangement. However, the overall integrity of chloroplasts was largely preserved, suggesting a relatively high level of structural stability. Mitochondria (M) retained their basic morphology, although minor changes in matrix density and cristae organization were observed, indicating stress-induced metabolic adjustments (Figure 6D). The general tissue organization was maintained, reflecting the ability of Murov 2 to preserve leaf anatomical integrity under elevated temperature. Under control conditions, mesophyll cells (Mc) of the Aran genotype exhibited a typical cellular organization with intact structural components (Figure 7A). Light microscopy observations confirmed a normal anatomical organization of leaf tissues, with a compact epidermal layer (E) and regularly arranged mesophyll (Mc) cells forming distinct intercellular spaces. Clearly visible stomata cells (St) (Figure 7A). In TEM electronograms, chloroplasts (Ch) showed a regular elongated shape, with well-developed thylakoid membranes (Th). The chloroplast envelope remained continuous, and the stroma appeared relatively homogeneous, indicating a normal physiological state. Mitochondria (M) displayed a characteristic oval morphology with clearly visible inner membranes and well-defined cristae. The cell wall (Cw) was continuous and mechanically stable. After exposure to heat stress, pronounced ultrastructural alterations were observed in the mesophyll cells of the Aran genotype (Figure 7B,D). Light microscopy demonstrated substantial anatomical disruptions in Aran leaves under heat stress. Mesophyll cells (Mc) became irregularly shaped, with enlarged intercellular spaces, indicating tissue loosening and reduced mechanical stability. In some regions, partial collapse of tissue structure was visible, while the epidermal layer (E) showed signs of deformation. Destructive changes were detected in the stomata (St) cells (Figure 7B). TEM images revealed severe damage to chloroplast (Ch) organization, including marked swelling, disorganization of thylakoid membranes (Th), partial loss of grana stacking, and increased electron translucency of the stroma, indicating impairment of the photosynthetic apparatus. In some cells, chloroplast envelopes appeared distorted, suggesting membrane destabilization. Mitochondria (M) exhibited structural abnormalities, including matrix loosening and partial disintegration of cristae, reflecting stress-induced disturbances in respiratory metabolism (Figure 7D). The cytoplasmic organization appeared less compact, and signs of cellular disorganization were evident.

## 3. Discussion

Heat stress directly disrupts cellular membranes by increasing fluidity, impairing lipid–protein interactions, and promoting oxidative damage, ultimately leading to enhanced permeability and electrolyte leakage. Accordingly, membrane stability indices are widely recognized as reliable physiological indicators of thermotolerance [30,31,32]. In the present study, the progressive increase in MDR and EL confirmed temperature-dependent membrane destabilization, while the parallel accumulation of MDA provided biochemical evidence that this injury was accompanied by lipid peroxidation. The lower MDR, EL, and MDA levels observed in Murov 2 and Zirva 85 compared with the sensitive genotypes suggest more effective protection against oxidative membrane damage. This is consistent with previous wheat studies showing that heat-tolerant genotypes generally maintain lower electrolyte leakage and reduced lipid peroxidation under elevated temperature [33,34,35]. Thus, membrane thermostability in tolerant genotypes appears to reflect an integrated protective response involving both antioxidant activity and heat-responsive molecular defense. The maintenance of membrane integrity is closely linked to antioxidant defense and the activity of molecular chaperones. The accumulation of sHSPs, such as HSP16.9, represents an important adaptive response that contributes to protein stabilization and the preservation of cellular homeostasis under heat stress [36,37]. Consistent with our results, previous studies have shown that *HSP16.9* is strongly induced in wheat upon heat exposure and that higher transcript levels are frequently associated with enhanced thermotolerance. Li et al. (2014) [15] reported that transcription of the wheat *TaHSP16.9* gene increased markedly after heat treatment at 42 °C, with heat-tolerant genotypes exhibiting higher expression levels than heat-sensitive cultivars. Their findings further indicated that regulatory elements located within the 3′-UTR region of the gene may enhance transcription under heat stress. Genetic studies have also demonstrated that variation at the *HSP16.9* locus contributes to phenotypic differences in heat tolerance in wheat. Garg et al. (2012) [38] identified a single-nucleotide polymorphism in the *HSP16.9* gene associated with differences in grain weight under terminal heat stress, suggesting that this gene may serve as a useful marker for selecting heat-tolerant wheat cultivars. The functional relevance of HSP16.9-type proteins has also been demonstrated in other cereals. In barley, suppression of *HvHSP16.9* significantly reduced stress tolerance, whereas higher expression levels were observed in tolerant genotypes, underscoring the protective role of *sHSPs* in maintaining cellular stability under stress conditions [39]. Therefore, the stronger induction of HSP16.9 detected in the present study in the tolerant genotypes Murov 2 and Zirva 85 is consistent with previous evidence that heat-tolerant plants are characterized by stronger activation of stress-responsive chaperone systems. sHSPs stabilize partially unfolded proteins and maintain them in a folding-competent state until normal cellular conditions are restored, thereby preventing irreversible protein aggregation and preserving metabolic functions under heat stress. In contrast, the lower induction of HSP16.9 observed in the more sensitive genotypes Aran and Gyzyl bugda may indicate a weaker molecular chaperone response. This interpretation is further supported by the densitometric quantification of HSP16.9 protein levels, which showed significant stress-induced accumulation in Murov 2 and Zirva 85, a significant but weaker response in Gyzyl bugda, and no statistically significant accumulation in Aran. The absence of significant protein accumulation in Aran despite a detectable transcript-level increase points to possible post-transcriptional limitations or accelerated protein turnover in this genotype, suggesting that the disconnect between mRNA and protein levels may represent an additional layer of vulnerability in heat-sensitive wheat. Under control conditions, HSP16.9 protein levels were low and in some genotypes below the visual detection threshold of the Western blot, but were confirmed by densitometric analysis, consistent with the known biology of small heat shock proteins as strictly stress-inducible chaperones expressed at minimal basal levels.

In the present study, genotype-dependent differences in *DREB* induction suggest that thermotolerance was associated not only with the magnitude of downstream protective responses, but also with the efficiency of their transcriptional coordination. This interpretation is supported by previous studies on *DREB*-mediated responses in wheat under heat and other abiotic stresses [40,41,42]. Genome-wide analysis of the wheat *DREB* family identified *TaDREB3* as a stress-responsive component, while functional characterization of *TaDREB3-A1* showed that its ectopic expression enhanced tolerance to heat, drought, and salinity, providing direct evidence that *DREB* activity in wheat can contribute to thermotolerance [41]. Similarly, genome-wide and transcriptome-based studies of *AP2/ERF* genes in contrasting wheat genotypes under heat stress identified heat-responsive candidates that clearly differentiated tolerant and sensitive genotypes [43]. Subsequent validation at the grain-filling stage further linked *TaAP2/ERF*-dependent heat tolerance with coordinated transcriptional programs associated with heat acclimation and the maintenance of seed development under stress conditions [44]. The presence of a *DRE*-related motif in the *HSP16.9* promoter is also consistent with the possible involvement of *DREB*-associated transcriptional regulation in *HSP16.9* induction under heat stress (Supplementary Figure S1). In this context, the stronger *DREB* induction observed in Murov 2 and Zirva 85 is unlikely to represent an isolated transcriptional event; rather, it probably reflects more efficient activation of upstream regulatory circuits that support the coordinated deployment of chaperone systems and redox defense under elevated temperature. Conversely, the weaker *DREB* response in Aran is consistent with its lower membrane stability, reduced HSP16.9 accumulation, and less coordinated antioxidant reprogramming.

The accumulation of ROS is one of the earliest consequences of heat stress in plants and, at the same time, an important signaling factor linking antioxidant responses with the activation of HSF-dependent chaperone systems, including HSP16.9 [45,46,47,48]. To limit excessive ROS accumulation, plants employ a multicomponent antioxidant system comprising SOD, CAT, and various peroxidases. SOD catalyzes the conversion of superoxide radicals into hydrogen peroxide and thus represents the first enzymatic barrier against oxidative stress, whereas CAT, GPX, and BPX detoxify hydrogen peroxide and other peroxides, thereby preventing oxidative damage to membranes, proteins, and nucleic acids [18,49]. Previous studies have shown that increased antioxidant enzyme activity plays a decisive role in plant thermotolerance. Enhanced activities of SOD, CAT, and peroxidases have been associated with improved membrane stability and reduced lipid peroxidation under heat stress [18,50], particularly during terminal heat stress, which is often accompanied by pronounced metabolic and transcriptional reprogramming [51]. However, the present data indicate that, in the studied material, antioxidant reprogramming was not uniform across all enzymes. The clearest distinction between tolerant and sensitive genotypes was observed for CAT and, especially, BPX, whereas total SOD activity was less informative. This suggests that, for this set of genotypes, the critical factor was not simply a general enhancement of the first step of ROS scavenging, but rather a more efficient subsequent detoxification of H_2_O_2_ and related peroxides.

These findings are also consistent with our previous study, in which we investigated the activity and isoenzyme composition of APX in the same wheat genotypes under heat stress. In that work, short-term heat stress caused a significant increase in APX activity in Zirva 85 and Murov 2, whereas Aran and Gyzyl bugda exhibited decreased enzyme activity. Electrophoretic analysis further revealed increased intensity of several APX isoforms in tolerant genotypes, indicating enhanced ROS-scavenging capacity under thermal stress [52]. Taken together, these observations support the hypothesis that the efficiency of the antioxidant defense system plays a critical role in determining the thermotolerance of wheat genotypes.

Analysis of SOD isoforms provided additional insight into compartment-specific redox regulation. Since *CuZnSOD* is localized in the cytosol and chloroplast stroma, *FeSOD* primarily in chloroplasts, and *MnSOD* in mitochondria, their responses reflect the regulation of distinct cellular compartments [18,53]. In the present study, *FeSOD* and *MnSOD* were the most informative isoforms for distinguishing tolerant and sensitive responses. Their more coordinated induction in Murov 2 and Zirva 85 indicates more effective antioxidant protection of chloroplasts and mitochondria, whereas the response in Aran and Gyzyl bugda was less balanced. Because chloroplasts are major sites of ROS generation under heat stress due to disruption of the photosynthetic electron transport chain, increased *FeSOD* expression is consistent with the activation of chloroplast antioxidant defense mechanisms [54]. It is particularly noteworthy that, in Aran, *CuZnSOD* induction was not accompanied by an effective overall SOD response and did not prevent pronounced physiological damage, indicating that induction of this single isoform was insufficient to provide full protection. Similarly, in Gyzyl bugda, increased *MnSOD* expression was accompanied by *FeSOD* suppression and the absence of significant changes in *CuZnSOD*, also indicating less integrated antioxidant reprogramming. Given the central role of chloroplastic *FeSOD* in detoxifying superoxide generated during photosynthesis, its suppression in sensitive genotypes may impair chloroplast protection and contribute to the reduction in total SOD activity. *MnSOD* is required for neutralizing superoxide radicals generated by the mitochondrial respiratory electron transport chain [49]. Although *MnSOD* transcription increased in all genotypes, enhanced coordination among all isoforms was observed predominantly in Murov 2 and Zirva 85, indicating integrated antioxidant regulation across the cytosolic, chloroplastic, and mitochondrial compartments. Similar genotype-dependent differences in the regulation of SOD isoforms have previously been described in wheat under abiotic stress [53] and are also consistent with the findings of Isgandarova et al. (2024) [55], who demonstrated the influence of genotype and ontogenetic context on isoform-specific responses. Overall, these findings confirm that antioxidant regulation under heat stress in wheat is not only genotype-dependent, but also compartment-specific.

The genotype-specific correlation matrices further supported the conclusion that thermotolerance is associated with coordinated rather than isolated stress responses. In the tolerant genotypes, *DREB* expression, *HSP16.9* transcript and protein accumulation, SOD isoform-related parameters, total SOD, BPX, CAT, and GPX were predominantly positively correlated, reflecting close integration between transcriptional regulation, chaperone-related protection, and antioxidant defense. The positive correlations between *HSP16.9* transcript and protein levels observed within genotypes indicate consistency between heat-induced transcriptional activation and protein accumulation. However, in the sensitive genotypes, *DREB-* and *HSP16.9*-related markers were not consistently accompanied by parallel positive associations with antioxidant enzymes. Instead, several antioxidant components, particularly total SOD, CAT, GPX, BPX, and *FeSOD*, showed weak or negative relationships with stress-responsive molecular markers, depending on genotype. This pattern indicates that, although heat-responsive genes were activated in sensitive genotypes, their activation was only partially coupled with antioxidant defense. Overall, these genotype-specific correlations reinforce the view that wheat thermotolerance is associated with the coordinated activation of transcriptional, chaperone-related, and redox-protective networks, whereas partial uncoupling of these responses may contribute to greater heat susceptibility.

Finally, the physiological, biochemical, and molecular differences between genotypes were supported by histological and ultrastructural observations. Heat stress caused structural alterations in leaf mesophyll cells; however, these changes were markedly less severe in the tolerant genotype Murov 2 than in the sensitive genotype Aran. This contrast indicates that heat tolerance is associated not only with stronger antioxidant and molecular stress responses, but also with a greater capacity to preserve cellular and organellar integrity under elevated temperature. In particular, the relative preservation of chloroplast and mitochondrial structure in Murov 2 is consistent with its lower membrane damage, stronger *HSP16.9* induction, more coordinated *DREB* response, and more efficient antioxidant reprogramming. Conversely, the pronounced structural disorganization observed in Aran supports its heat-sensitive phenotype and suggests that insufficient molecular and redox protection is accompanied by destabilization of photosynthetic and respiratory organelles. Recent studies have similarly shown that stress-induced anatomical and ultrastructural changes in leaves are informative indicators of genotype-dependent tolerance under abiotic stresses, including salinity and drought [56,57,58]. Since chloroplasts and mitochondria are membrane-rich organelles that are highly vulnerable to heat-induced oxidative injury, their structural preservation appears to be an important component of thermotolerance. The disorganization of thylakoid membranes observed in sensitive genotypes under heat stress is likely to impair not only photosynthetic electron transport but also regulatory mechanisms such as energy-dependent quenching, which depend on the structural integrity of the thylakoid membrane system [59]. Overall, the contrasting ultrastructural responses of Murov 2 and Aran support the broader conclusion that wheat heat tolerance depends on coordinated protection at the membrane, organelle, antioxidant, and molecular levels.

## 4. Materials and Methods

*Plant material and growing conditions*

Four local winter bread wheat genotypes Murov 2 and Zirva 85, tolerant to abiotic stress factors, Aran and Gyzyl bugda, stress-sensitive genotypes were selected from the wheat Gene Fund of the Research Institute of Crop Husbandry (Azerbaijan). To increase the reliability of the expression analysis, the experiments were conducted in randomized complete blocks, in 2 variants, 10 biological and 3 technical replicates. The plants were grown in an artificial climate chamber with a light/dark period of 16/8 h and 24 °C/18 °C day/night temperature regime, relative humidity of 50%, in individual plastic cups, in a soil–sand mixture (1/3 ratio) and 14-day-old seedlings were exposed to stress. Heat stress was created in the laboratory in a thermostat at 38 °C for 30 min, 40 °C for 30 min, 42 °C for 2 h. The leaf samples were immediately frozen in liquid nitrogen and stored at −80 °C until analysis.

*Membrane damage rate (MDR)*

Leaf samples of seedlings (250 mg), were placed into Erlenmeyer flasks containing 25 mL of distilled water and heated in a water bath for 5 min, with the temperature increased at 1 °C intervals. This treatment regime was selected according to methodological recommendations indicating that short-term exposure to high temperatures serves as a test for the primary thermostability of plant cells. Leaves without heat treatment were used as control samples. After heating, the flasks were incubated on a laboratory shaker in water for 2 h. To determine the total electrolyte leakage (EL) from tissues (taken as 100%), the flasks with samples were then placed in a boiling water bath for 30 min. After cooling, membrane permeability was evaluated based on the conductivity of aqueous extracts measured using a Horiba Scientific conductivity meter (HORIBA Advanced Techno Co., Ltd., Kyoto, Japan). Membrane permeability was expressed as the percentage of electrolyte concentration in the extract relative to the total electrolyte release. EL values were used to calculate MDR according to the following formula [60]:

$$MDR=\frac{L_{D}-L_{0}}{100-L_{0}}\times 100(\%)$$

*L_D_*—electrolyte leakage from heat-treated tissue, % of total leakage.

*L*_0_—electrolyte leakage from control tissue, % of total leakage.

*Lipid Peroxidation Assay*

The intensity of lipid peroxidation was assessed by measuring malondialdehyde (MDA) content according to the method of Heath and Packer (1968) [61]. MDA concentration was quantified spectrophotometrically using a Thermo Scientific Evolution 350 UV/Vis spectrophotometer (Thermo Fisher Scientific, Waltham, MA, USA) based on its reaction with 0.5% thiobarbituric acid. Absorbance values were recorded at 532 and 600 nm. The MDA content was calculated according to the following formula:

$$A=\frac{D_{532}-D_{600}}{E\times m}$$

where *A* is the MDA concentration, *D* is the absorbance value, *m* is fresh biomass, and *E* is the extinction coefficient.

*Protein Extraction and Quantification*

Frozen leaf tissue (~200 mg per sample) was ground to a fine powder in liquid nitrogen using a pre-chilled mortar and pestle. The powder was homogenized in 500 µL of ice-cold extraction buffer (50 mM Tris–HCl, pH 7.5, 150 mM NaCl, 1 mM EDTA) supplemented immediately before use with 1 mM phenylmethylsulfonyl fluoride (PMSF) and 2 mM dithiothreitol. The homogenate was centrifuged at 14,000× *g* for 15 min at 4 °C. The clarified supernatant, containing total soluble protein, was collected for quantification and downstream analysis. All experiments included at least three independent biological replicates. Protein concentration was determined using the Bradford assay [62] with bovine serum albumin (BSA) as a standard. Appropriate volumes of protein samples and standards were mixed with Bradford reagent containing Coomassie Brilliant Blue G-250 (0.01% *w*/*v*), ethanol (5% *v*/*v*), and phosphoric acid (10% *v*/*v*), and incubated at room temperature for 15 min. Absorbance was measured at 595 nm using a spectrophotometer, and protein concentrations were calculated from the BSA standard curve and expressed as µg/µL.

*SDS–PAGE and Immunoblotting*

Equal amounts of protein (25 µg per lane) were mixed with Laemmli sample buffer and denatured at 95 °C for 5 min. Proteins were separated by SDS-polyacrylamide gel electrophoresis (SDS–PAGE) on a 15% polyacrylamide gel containing 6 M urea [63]. Following electrophoresis, proteins were transferred onto PVDF membrane using an iBlot™ 2 dry blotting system (Program P1; Thermo Fisher Scientific). Membrane was blocked in 5% (*w*/*v*) non-fat dry milk in Tris-buffered saline containing 0.1% Tween-20 for 1 h at room temperature, followed by overnight incubation at 4 °C with a polyclonal anti-HSP16.9 antibody (AS12 2570; Agrisera, Vännäs, Sweden) diluted 1:2000 in TBST. Immunoreactive signals were detected using Clarity™ Western ECL substrate (Thermo Fisher Scientific Waltham, MA, USA) and visualized with an iBright™ FL1000 Imaging System (Thermo Fisher Scientific Waltham, MA, USA). Band intensities were quantified by densitometric analysis using ImageJ (NIH, Bethesda, MD, USA) and normalized to total protein loaded per lane.

*Gene Expression Analysis*
*RNA Extraction and cDNA Synthesis*

Total RNA was extracted from leaf material using a Monarch Total RNA Miniprep Kit (New England Biolabs, Ipswich, MA, USA) following the manufacturer’s instructions. Genomic DNA contamination was removed using RNase-free DNase I. The quality and quantity of the extracted RNA were assessed by agarose gel electrophoresis. RNA concentration was measured spectrophotometrically using a NanoDrop 2000C Spectrophotometer (Thermo Scientific, Waltham, MA, USA). Single-stranded cDNA synthesis was performed from the total RNA using a LunaScript RT SuperMix Kit (New England Biolabs, Ipswich, MA, USA) according to the manufacturer’s instructions, in a final volume of 20 µL.

*Quantitative Real-Time PCR*

PCR was conducted using a Mic Real-Time PCR system (Bio Molecular Systems, Upper Coomera, QLD, Australia) in a total reaction volume of 20 µL. Each reaction mixture contained 10 µL of Luna Universal qPCR Mix (New England Biolabs, Ipswich, MA, USA), 1 µL of 1:5 diluted cDNA, 0.5 µL each of forward and reverse primers (10 µM), and 7 µL of nuclease-free water. The PCR protocol included an initial denaturation step at 94 °C for 60 s, followed by 45 cycles of 95 °C for 15 s and 60 °C for 30 s. No-template controls (NTCs) were included for each primer pair. Each reaction was performed in triplicate (technical replicates) for each of the three biological replicates. Elongation factor 1 alpha (Elf1-α) was chosen as a housekeeping gene due to its stable and consistent expression across different experimental conditions. The primer sequences used for expression analysis are listed in Supplementary Table S4. Primer efficiency for each pair was determined by the standard curve method using serial dilutions of cDNA, calculated using the formula efficiency:

$$\mathrm{Efficiency}\;\left(\%\right)=\left(10^{-\frac{1}{\mathrm{slope}}}-1\right)\times 100$$

Dissociation curves for each amplicon were analyzed to confirm the specificity of the amplification reactions. Fold change in gene expression (stressed versus control) was calculated using the 2^−∆∆Ct^ method [64].

*Enzyme Activity Measurement*
*Isolation of the enzyme extract*

Leaf samples (0.5 g) were crushed in liquid nitrogen and dissolved in a buffer solution containing 100 mM Na-phosphate (pH 7.8), 1 mM EDTA, 2 mM PMSF, 1% polyvinylpyrrolidone and 0.1% Triton X-100 (Sigma-Aldrich, St. Louis, MO, USA). The homogenized samples were then centrifuged at 15,000× *g* for 20 min at 4 °C. The resulting supernatant was used to determine the activity of antioxidant enzymes.

*Superoxide Dismutase (SOD, EC 1.15.1.1)*

To determine the activity of SOD enzyme, SOD Assay Kit-WST (Sigma-Aldrich, St. Louis, MO, USA) was employed. In this study, the cytosolic isoform of SOD from plant cells was analyzed. Leaves were homogenized in 50 mM potassium phosphate buffer (pH 7.8) and the homogenate was centrifuged. The optical density of the reaction was measured at a wavelength of 450 nm to assess SOD activity.

*Guaiacol Peroxidase (GPX, EC 1.11.1.7)*

GPX activity was quantified spectrophotometrically by monitoring the increase in absorbance at 470 nm over a 2 min interval [65]. The reaction mixture contained 50 mM sodium phosphate buffer (pH 7.0), 25 mM guaiacol, 25 mM H_2_O_2_, and 20 µL of enzyme extract. Enzyme activity was calculated using a molar extinction coefficient (ε) of 26.6 mM^−1^ cm^−1^ and expressed as µmol guaiacol oxidized per mg protein per minute.

*Benzidine Peroxidase (BPX, EC 1.11.1.7)*

BPX activity was determined by measuring the increase in absorbance at 590 nm for 1 min following the addition of enzyme extract to the reaction system [66]. Activity values were calculated using an extinction coefficient (ε) of 39 mM^−1^ cm^−1^ and expressed as µmol benzidine oxidized per mg protein per minute.

*Catalase (CAT, EC 1.11.1.6)*

Catalase activity was assessed by monitoring the decomposition rate of hydrogen peroxide at 240 nm. Briefly, 1 g of flag leaf tissue was homogenized in 50 mM potassium phosphate buffer (pH 7.0). The homogenate was filtered and centrifuged at 8000× *g* for 10 min, and the resulting supernatant was used for enzymatic analysis. The reaction mixture consisted of 2.9 mL phosphate buffer and 25 µL enzyme extract. The reaction was initiated by adding 90 µL of 3% H_2_O_2_. The decrease in absorbance at 240 nm was recorded per minute. Enzyme activity was calculated using a molar extinction coefficient (ε) of 39.4 mM^−1^ cm^−1^ and expressed as µmol H_2_O_2_ decomposed per mg protein per minute [67].

*Analysis of Leaf Mesophyll Cells by Light and Transmission Electron Microscopy (TEM)*

Plant samples were fixed in a solution containing 2% paraformaldehyde, 2% glutaraldehyde, 1% caffeine, and 0.1% picric acid prepared in 0.1 M phosphate buffer (pH 7.4). After the samples were kept in the fixative for at least 24 h, they were post-fixed for two hours in a 1% osmium tetroxide solution prepared in phosphate buffer (pH 7.4). Araldite–Epon blocks were prepared from the material according to standard protocols accepted in electron microscopy [68]. Semi-thin sections (1–2 µm) obtained from the blocks using an EM UC7 (Leica, Microsystems, Buffalo Grove, IL, USA) ultramicrotome were stained by a one-step polychromatic method [69], examined under a Primo Star (Carl Zeiss, Oberkochen, Germany) microscope, and images of the required areas were captured using an EOS D650 (Canon, Inc., Tokyo, Japan) digital camera. Ultrathin sections (50–70 nm) obtained from the same blocks were first stained with a 2% uranyl acetate solution and then with 0.6% lead citrate prepared in 0.1 N NaOH solution. The ultrathin sections were examined using a JEM-1400 (JEOL, Ltd., Tokyo, Japan) transmission electron microscope at an accelerating voltage of 80–120 kV, and electron micrographs were obtained. Morphometric analysis of TIFF-format electron micrographs was conducted using The TEM Imaging Platform (version 5.2, Build 3554), developed by Olympus Soft Imaging Solutions GmbH (Münster, Germany).

*Statistical analysis*

Statistical analysis was performed in R version 4.5.3 (R Foundation for Statistical Computing, Vienna, Austria). Data were expressed as mean ± standard error (SE) from at least three independent biological replicates. Differences between control and stress-treated samples were assessed using two-way analysis of variance (ANOVA), followed by Tukey’s honestly significant difference (HSD) post hoc test for multiple comparisons. Statistical significance of Pearson correlation coefficients was assessed using the t-distribution-based test implemented in R’s cor.test function (two-tailed). Significance thresholds were set at * *p* < 0.05, ** *p* < 0.01, and *** *p* < 0.001.

## 5. Conclusions

The present study demonstrates that heat tolerance in bread wheat is an integrated multilevel trait involving membrane stability, antioxidant defense, stress-responsive gene expression, molecular chaperone accumulation, and preservation of cellular ultrastructure. Heat stress induced membrane destabilization, oxidative imbalance, and structural injury in all genotypes; however, the tolerant genotypes Murov 2 and Zirva 85 retained a higher protective capacity than the sensitive genotypes Aran and Gyzyl bugda. Their greater thermotolerance was associated with reduced membrane injury, enhanced CAT- and BPX-mediated peroxide detoxification, more balanced regulation of *FeSOD* and *MnSOD* expression, and stronger induction of *DREB* and *HSP16.9.* HSP16.9 protein accumulated significantly under heat stress in tolerant genotypes and in Gyzyl bugda, while the lack of significant accumulation in Aran despite detectable transcript induction may indicate genotype-specific post-transcriptional constraints. Genotype-specific Pearson correlation analyses further showed that wheat thermotolerance was associated with greater coordination among transcriptional, chaperone-related, and antioxidant responses, whereas heat-sensitive genotypes exhibited more heterogeneous and partially uncoupled relationships among these markers. Ultrastructural observations confirmed that thermotolerance is associated with improved preservation of chloroplast, mitochondrial, and mesophyll organization under elevated temperature.

Overall, the findings indicate that wheat adaptation to heat stress depends on the effective integration of membrane integrity, redox homeostasis, proteostasis, and organelle stability. These traits may serve as useful physiological and molecular indicators for screening and breeding heat-tolerant wheat genotypes. However, the observed associations between *DREB* and *HSP16.9* induction and thermotolerance-related traits remain primarily correlative. Future functional validation through overexpression, knockdown/gene-silencing, or CRISPR/Cas-based approaches will be necessary to confirm the direct causal roles of these genes in wheat thermotolerance. In addition, validation of the identified physiological and molecular indicators under greenhouse and field conditions, as well as under combined heat–drought stress, will be important to confirm their agronomic relevance for wheat improvement programs.

## Data Availability

The data supporting the findings of this study are available within the article and its Supplementary Materials. Additional raw data may be made available from Samira Rustamova (s.rustamova@imbb.science.az) upon reasonable request.

## Supplementary Materials

The following supporting information can be downloaded at https://www.mdpi.com/article/10.3390/plants15121896/s1, Supplementary Table S1: Electrolyte leakage (EL, %) in wheat genotypes under heat stress (49–57 °C). Values represent mean ± SE (n = 3); Supplementary Table S2: Two-way ANOVA results for the effects of genotype, heat treatment, and their interaction on antioxidant enzyme activities and stress-responsive gene expression traits in wheat genotypes; Supplementary Table S3: In silico transcriptomic evidence supporting heat-responsive expression of HSP16.9- and DREB-related genes in wheat; Supplementary Table S4: Sequences of primers used for qRT-PCR; Supplementary Figure S1: In silico identification of a putative DREB-responsive cis-element in the upstream regulatory region of HSP16.9.

## Abbreviations

The following abbreviations are used in this manuscript:

APX	Ascorbate peroxidase
BPX	Benzidine peroxidase
CAT	Catalase
CuZnSOD	Copper/zinc superoxide dismutase
DREB	Dehydration-responsive element-binding protein
DRE	Dehydration-responsive element
EL	Electrolyte leakage
FeSOD	Iron superoxide dismutase
GPX	Guaiacol peroxidase
HSF	Heat shock transcription factor
HSP16.9	16.9 kDa heat shock protein
MDA	Malondialdehyde
MDR	Membrane damage rate
MnSOD	Manganese superoxide dismutase
sHSP	Small heat shock protein
SOD	Superoxide dismutase
TEM	Transmission electron microscopy
