# Supplementary material for: Integrated Assessment of Physiological, Molecular and Ultrastructural Responses to Heat Stress in Wheat
Source: Plants (Basel). 2026 Jun 18;15(12):1896. doi: 10.3390/plants15121896 (PMC13306779; doi:10.3390/plants15121896)
Supplement: Supplementary file 1 [file plants-15-01896-s001.zip › plants-4322356-supplementary.pdf]

Supplementary Table S1. Electrolyte leakage (EL, %) in wheat genotypes under heat stress (49–57 °C). Values represent mean ± SE (n = 3).

Temperature (°C)	Aran	Gyzyl bugda	Murov 2	Zirva 85
**49**	57.93 ± 0.66	47.93 ± 0.66	45.23 ± 0.49	42.32 ± 0.52
**50**	59.07 ± 0.75	52.27 ± 0.84	44.45 ± 0.50	44.13 ± 0.55
**51**	63.73 ± 0.78	56.37 ± 0.84	46.34 ± 0.50	44.34 ± 0.66
**52**	71.03 ± 0.90	61.20 ± 0.92	52.67 ± 0.53	48.56 ± 0.81
**53**	77.97 ± 0.84	67.97 ± 0.90	55.45 ± 0.55	52.23 ± 0.81
**54**	85.97 ± 0.95	74.20 ± 0.92	58.34 ± 0.46	55.54 ± 0.90
**55**	91.97 ± 0.90	80.30 ± 0.92	62.23 ± 0.66	58.45 ± 0.90
**56**	95.97 ± 0.95	85.57 ± 0.90	66.35 ± 0.80	61.13 ± 0.84
**57**	97.90 ± 0.84	89.97 ± 0.95	72.36 ± 0.66	64.34 ± 0.84

Supplementary Table S2. Two-way ANOVA results for the effects of genotype, heat treatment, and their interaction on antioxidant enzyme activities and stress-responsive gene expression traits in wheat genotypes.

Parameter	Source of variation	df	Sum of squares	Mean square	F-value	p-value
BPX	Genotype	3	128.718	42.906	365.157	6.12 x 10^-15
BPX	Treatment	1	46.760	46.760	397.961	9.95 x 10^-13
BPX	Genotype x Treatment	3	76.191	25.397	216.145	3.73 x 10^-13
BPX	Residuals	16	1.880	0.118	-	-
CAT	Genotype	3	567.321	189.107	1319.352	2.30 x 10^-19
CAT	Treatment	1	1.260	1.260	8.794	0.0091
CAT	Genotype x Treatment	3	248.235	82.745	577.290	1.64 x 10^-16
CAT	Residuals	16	2.293	0.143	-	-
CuZnSOD	Genotype	3	1642.825	547.608	3361.278	1.32 x 10^-22
CuZnSOD	Treatment	1	88.550	88.550	543.532	8.89 x 10^-14
CuZnSOD	Genotype x Treatment	3	28.625	9.542	58.567	7.56 x 10^-9
CuZnSOD	Residuals	16	2.607	0.163	-	-
DREB	Genotype	3	19.531	6.510	50.241	2.30 x 10^-8
DREB	Treatment	1	131.134	131.134	1011.965	6.81 x 10^-16
DREB	Genotype x Treatment	3	23.041	7.680	59.270	6.93 x 10^-9
DREB	Residuals	16	2.073	0.130	-	-
FeSOD	Genotype	3	20.616	6.872	71.198	1.80 x 10^-9
FeSOD	Treatment	1	1.712	1.712	17.737	0.0007
FeSOD	Genotype x Treatment	3	23.847	7.949	82.357	6.08 x 10^-10
FeSOD	Residuals	16	1.544	0.097	-	-

GPX	Genotype	3	1894.880	631.627	1288.760	2.77 x 10^-19
GPX	Treatment	1	3.338	3.338	6.810	0.0190
GPX	Genotype x Treatment	3	231.099	77.033	157.177	4.43 x 10^-12
GPX	Residuals	16	7.842	0.490	-	-
HSP16.9	Genotype	3	205.166	68.389	506.520	4.62 x 10^-16
HSP16.9	Treatment	1	441.870	441.870	3272.707	6.18 x 10^-20
HSP16.9	Genotype x Treatment	3	205.070	68.357	506.283	4.63 x 10^-16
HSP16.9	Residuals	16	2.160	0.135	-	-
MnSOD	Genotype	3	37.108	12.369	31.725	5.74 x 10^-7
MnSOD	Treatment	1	155.754	155.754	399.472	9.67 x 10^-13
MnSOD	Genotype x Treatment	3	43.910	14.637	37.539	1.81 x 10^-7
MnSOD	Residuals	16	6.238	0.390	-	-
SOD	Genotype	3	22.550	7.517	23.862	3.79 x 10^-6
SOD	Treatment	1	0.482	0.482	1.529	0.2341
SOD	Genotype x Treatment	3	13.348	4.449	14.125	9.25 x 10^-5
SOD	Residuals	16	5.040	0.315	-	-
MDA	Genotype	3	228.045	76.015	145.136	8.184 x 10^−12
MDA	Treatment	1	535.815	535.815	1023.036	6.251 x 10^−16
MDA	Genotype x Treatment	3	158.925	52.935	101.145	1.292 x 10^−10
MDA	Residuals	16	8.38	0.523	-	-
HSP16.9 protein accumulation	Genotype	3	0.698	0.232	13.117	0.0001
HSP16.9 protein accumulation	Treatment	1	1.539	1.540	86.731	7.325 x 10^−8
HSP16.9 protein accumulation	Genotype x Treatment	3	0.531	0.177	9.983	0.0005
HSP16.9 protein accumulation	Residuals	16	0.284	0.0177	-	-

Note: df, degrees of freedom; BPX, benzidine peroxidase; CAT, catalase; CuZnSOD, copper/zinc superoxide dismutase; DREB, dehydration-responsive element-binding protein; FeSOD, iron superoxide dismutase; GPX, guaiacol peroxidase; HSP16.9, 16.9 kDa heat shock protein; MnSOD, manganese superoxide dismutase; SOD, superoxide dismutase. p-values were obtained from two-way ANOVA.

Supplementary Table S3. In silico transcriptomic evidence supporting heat-responsive expression of HSP16.9- and DREB-related genes in wheat.

Gene ID	Putative annotation	Genome	Dataset	Comparison	Stage	log_2FC	Approx. fold change	Adjusted p-value
TraesCS3D02G045600	16.9 kDa class I heat shock protein 2-like	D	E-MTAB-8520	Warm/hot temperature regimen vs control	10 days post anthesis	5.8	55.7-fold	3.13×10^{-14}
TraesCS3D02G045600	16.9 kDa class I heat shock protein 2-like	D	E-MTAB-8520	Warm/hot temperature regimen vs control	14 days post anthesis	4.4	21.1-fold	1.56×10^{-13}
TraesCS3A02G099200	DREB-related transcription factor	A	E-MTAB-8520	Warm/hot temperature regimen vs control	14 days post anthesis	1.7	3.25-fold	1.26×10^{-5}

Note: Differential expression data were retrieved from Expression Atlas, EMBL-EBI, dataset E-MTAB-8520, “Effect of heat stress on developing wheat grain”. Gene annotations were checked using Ensembl Plants and WikiGene.

Supplementary Table S4. Sequences of primers used for qRT-PCR.

Target gene	Direction	Sequence (5′–3′)	Reference
MnSOD	F	AACATCTGGAAGGTGGTGAACT	[55]
	R	AACTCAAGAGCGAGCGAAGTA	
CuZnSOD	F	CTCCATGAGTTCGGTGACAT	
	R	GACGGACTTCATCTTCTGGT	
FeSOD	F	GAATTCCACTGGGGAAGCATC	
	R	GTAAGCGTGCTCCCAAACGTC	
HSP16.9	F	ATGTCGATCGTGAGGCGG	[60]
	R	TCAGCCGGAGATCTGGATG	
Elf1-α	F	CAGATTGGCAACGGCTACG	[40]
	R	CGGACAGCAAAACGACCAAG	
DREB	F	CTGTGGCTTGGTTCATTCCC	
	R	ACTCCGATTCATCCTTCCCA	

Supplementary Figure S1. In silico identification of a putative DREB-responsive cis-element in the upstream regulatory region of HSP16.9.

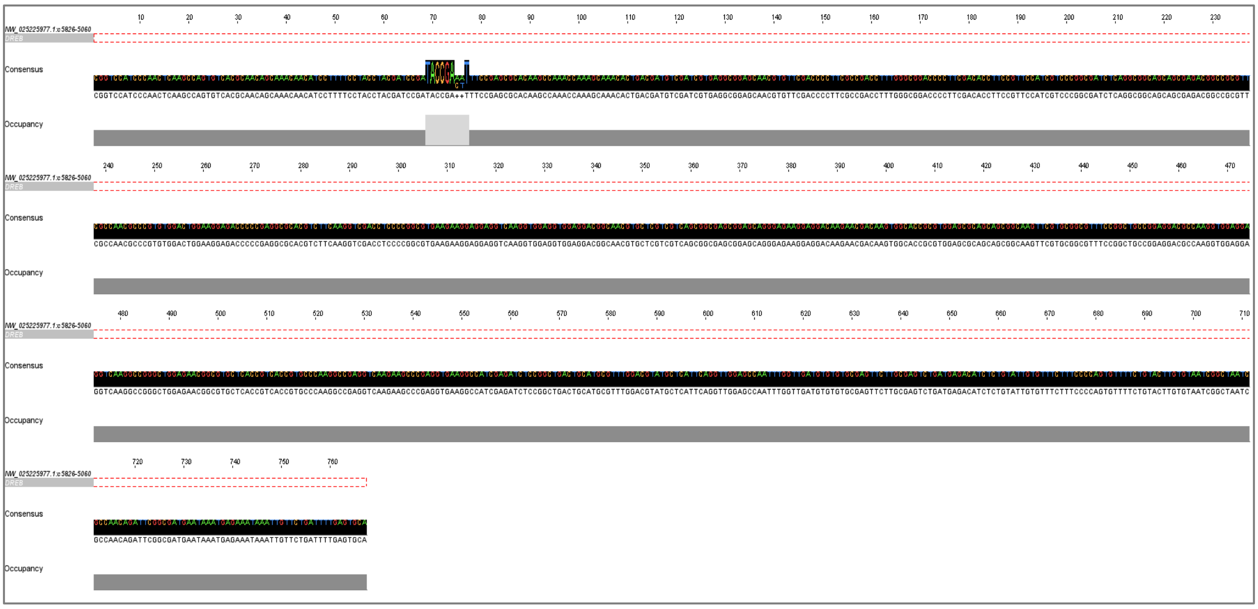


```
CLUSTAL format alignment by MAFFT (v7.511)

NW_0252259      cggtccatcccaactcaagccagtgtcacgcaacagcaaacaacatccttttcctaccta
DREB            ------------------------------------------------------------

NW_0252259      cgatccgataccgaattttccgagcgcacaagccaaaccaaagcaaacactgacgatgtc
DREB            ---------taccgacat------------------------------------------
                         ******   *

NW_0252259      gatcgtgaggcggagcaacgtgttcgaccccttcgccgacctttgggcggaccccttcga
DREB            ------------------------------------------------------------

NW_0252259      caccttccgttccatcgtcccggcgatctcaggcggcagcagcgagacggccgcgttcgc
DREB            ------------------------------------------------------------

NW_0252259      caacgcccgtgtggactggaaggagacccccgaggcgcacgtcttcaaggtcgacctccc
DREB            ------------------------------------------------------------

NW_0252259      cggcgtgaagaaggaggaggtcaaggtggaggtggaggacggcaacgtgctcgtcgtcag
DREB            ------------------------------------------------------------

NW_0252259      cggcgagcggagcagggagaaggaggacaagaacgacaagtggcaccgcgtggagcgcag
DREB            ------------------------------------------------------------

NW_0252259      cagcggcaagttcgtgcggcgtttccggctgccggaggacgccaaggtggaggaggtcaa
DREB            ------------------------------------------------------------

NW_0252259      ggccgggctggagaacggcgtgctcaccgtcaccgtgcccaaggccgaggtcaagaagcc
DREB            ------------------------------------------------------------

NW_0252259      cgaggtgaaggccatcgagatctccggctgactgcatgcgtttggacgtatgctcattca
DREB            ------------------------------------------------------------

NW_0252259      ggttggagccaatttggttgatgtgtgtgcgagttcttgcgagtctgatgagacatctct
DREB            ------------------------------------------------------------

NW_0252259      gtattgtgtttctttccccagtgttttctgtacttgtgtaatcggctaatcgccaacaga
DREB            ------------------------------------------------------------

NW_0252259      ttcggcgatgaataaatgagaaataaattgttctgattttgagtgca
DREB            -----------------------------------------------
```
